# Unforeseen Costs of Cutting Mosquito Surveillance Budgets

**DOI:** 10.1371/journal.pntd.0000858

**Published:** 2010-10-26

**Authors:** Gonzalo M. Vazquez-Prokopec, Luis F. Chaves, Scott A. Ritchie, Joe Davis, Uriel Kitron

**Affiliations:** 1 Department of Environmental Studies, Emory University, Atlanta, Georgia, United States of America; 2 Programa de Investigación en Enfermedades Tropicales, Escuela de Medicina Veterinaria, Universidad Nacional, Heredia, Costa Rica; 3 Cairns Tropical Public Health Unit, Queensland Health, Cairns, Queensland, Australia; 4 School of Public Health, Tropical Medicine and Rehabilitation Sciences, James Cook University, Cairns, Queensland, Australia; Centers for Disease Control and Prevention, United States of America

## Abstract

A budget proposal to stop the U.S. Centers for Disease Control and Prevention (CDC) funding in surveillance and research for mosquito-borne diseases such as dengue and West Nile virus has the potential to leave the country ill-prepared to handle new emerging diseases and manage existing ones. In order to demonstrate the consequences of such a measure, if implemented, we evaluated the impact of delayed control responses to dengue epidemics (a likely scenario emerging from the proposed CDC budget cut) in an economically developed urban environment. We used a mathematical model to generate hypothetical scenarios of delayed response to a dengue introduction (a consequence of halted mosquito surveillance) in the City of Cairns, Queensland, Australia. We then coupled the results of such a model with mosquito surveillance and case management costs to estimate the cumulative costs of each response scenario. Our study shows that halting mosquito surveillance can increase the management costs of epidemics by up to an order of magnitude in comparison to a strategy with sustained surveillance and early case detection. Our analysis shows that the total costs of preparedness through surveillance are far lower than the ones needed to respond to the introduction of vector-borne pathogens, even without consideration of the cost in human lives and well-being. More specifically, our findings provide a science-based justification for the re-assessment of the current proposal to slash the budget of the CDC vector-borne diseases program, and emphasize the need for improved and sustainable systems for vector-borne disease surveillance.

## Author Summary

Surveillance has served as a basis for important public health responses to new threats, and as a source of invaluable information for health providers and policy makers. A budget proposal to stop the U.S. Centers for Disease Control and Prevention (CDC) funding in surveillance and research for mosquito-borne diseases such as dengue and West Nile virus has the potential to leave the country ill-prepared to handle new emerging diseases and manage existing ones. The present article uniquely integrates infectious disease models with economic analysis, taking advantage of a unique detailed dataset. By coupling a mathematical model with cost analysis we were able to evaluate the impact of delayed control responses to dengue fever, a mosquito-transmitted disease of global importance, in an economically developed urban environment. Our analysis clearly shows that the total costs of preparedness through surveillance are far lower than the ones that follow the introduction of vector-borne pathogens. Our findings will help provide a science-based justification for re-assessment of the current proposal to slash the budget of the CDC vector-borne diseases program. More generally our study demonstrates the power of rigorous analysis of carefully collected data for a balanced assessment of the economic implications of a public health program shift.

## Introduction

The 2011 U.S. fiscal year proposed budget cut of $26.7 million from the CDC vector-borne diseases program [1] could virtually paralyze surveillance and research activities directed at diseases already circulating in the U.S such as dengue and West Nile virus (WNV), and jeopardize the capability of the existing health infrastructure for early detection of other exotic mosquito-transmitted pathogens such as Rift Valley fever, Japanese encephalitis and chikungunya virus [1]. Surveillance is the first line of defense against infectious diseases [2], guides health agencies' response to infectious threats, optimizes resources by focusing interventions on target areas, and generates invaluable information for health providers and policy makers [2]. We present here a case study where we couple a mathematical model with cost analysis to evaluate the economic impact of different response scenarios to the introduction of a vector-transmitted pathogen of public health importance into an economically developed urban environment.

## Methods

Data from two well-documented and successfully controlled dengue fever outbreaks introduced by viremic travelers into the city of Cairns, Queensland, Australia in 2003 and 2009 [3], [4] were used to derive the basic reproductive number (*R_0_*) and the effective reproduction number (*R_t_*) of dengue transmission. *R_0_* represent the average number of secondary cases after the introduction of an infection, and was estimated by fitting an exponential function to the observed weekly epidemic curves before vector control interventions began (6 weeks in 2003 and 4 weeks in 2009, Figure 1 A–B) following the method of Nishiura et al. [5]. *R_t_* represent the average number of secondary cases per primary case at time *t* of each outbreak and was estimated by accumulating the number of cases in biweekly periods (the average generation time of dengue is ∼14 days) and computing the ratio between consecutive two-week periods.

**Figure 1:**
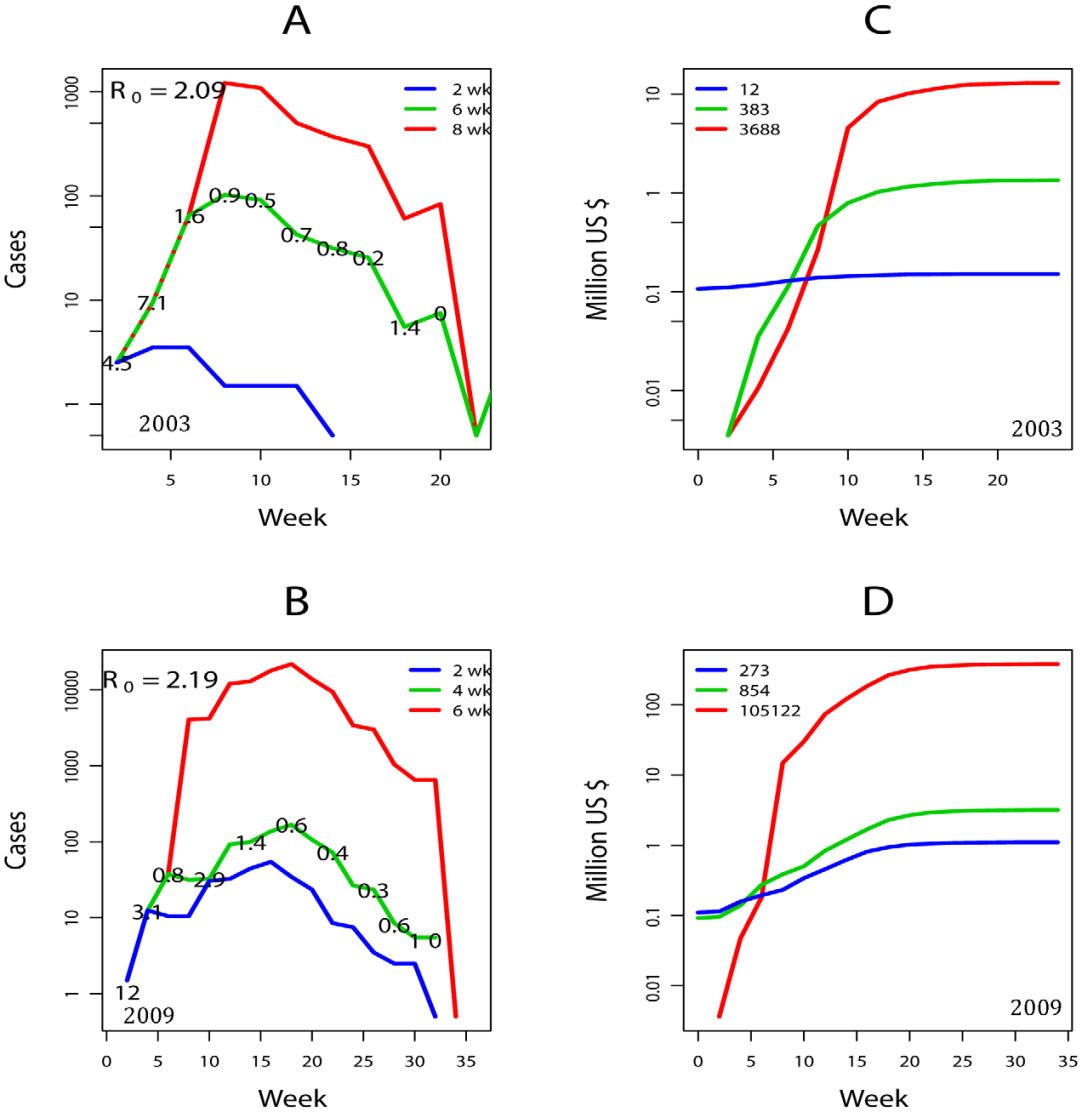
Impacts of hypothetical scenarios of delayed response of vector control to Dengue virus outbreaks. The basic reproduction number, *R_0_* (the average number of secondary cases after the introduction of an infection) for the 2003 and 2009 dengue fever outbreaks that affected the city of Cairns, Australia, was estimated by fitting an exponential function to the observed weekly epidemic curves before vector control interventions began (6 weeks in 2003 and 4 weeks in 2009). The effective reproduction number, *R_t_* (the average number of secondary cases per primary case at time *t*) of each outbreak was estimated by accumulating the number of cases in biweekly periods (the average generation time of dengue is ∼14 days) and computing the ratio between consecutive two-week periods. The hypothetical epidemic curves for the 2003 (**A**) and 2009 (**B**) outbreaks under different scenarios for response times (*res*) of vector control activities to a dengue introduction (*res* = 2, 4, 6 and 8 weeks) were computed by estimating the number of cases in the absence of control (between *t_0_* and *res*) using *R_0_*, and then generating the rest of each epidemic time series by multiplying the number of cases by the estimated post intervention *R_t_* in the original series. Blue lines indicate a faster response time than in the actual outbreak, red lines indicate scenarios where the response is delayed in comparison to the actual outbreak, and green lines indicate the actual outbreak. Values on top of the green lines are estimates for *R_t_*. Cumulative cost (in 2009 US$) of each *res* scenario were estimated for the 2003 (**C**) and 2009 (**D**) outbreaks. Figure legends refer to each *res* scenario (A,B) and to the final epidemic size of each scenario (C,D).

Hypothetical epidemic curves for the 2003 (Figure 1A) and 2009 (Figure 1B) outbreaks under different scenarios for response times (*res*) of vector control activities to a dengue introduction (*res* = 2, 4, 6 and 8 weeks) were computed by estimating the number of cases in the absence of control (between *t_0_* and *res*) using *R_0_*, and then generating the rest of each epidemic time series by multiplying the number of cases by the estimated post intervention *R_t_* in the original series. A response within 2 weeks of an introduction was assumed to occur only when active vector surveillance was in place (incurring a continuous cost), whereas delays in response of 6–8 weeks occurred when active disease and vector surveillance were eliminated.

Direct and indirect costs per case provided by the Cairns Public Health Unit of Queensland Health, Australia (Table 1), were used to estimate the cumulative cost (in 2009 US$) of each hypothetical response scenario (Figure 1 C–D). Costs were transformed from AU$ to US$ using each year's average exchange rate, and from 2003 US$ to 2009 US$ values using the Gross Domestic Product (GDP) deflator [6]. Direct costs included vector control, case diagnosis and blood bank screening costs, whereas indirect costs included work days lost due to disease. Our analysis assumed absence of dengue hemorrhagic fever manifestations (consequence of the lack of co-circulation of other dengue virus strains in epidemic settings) and no costs associated with subclinical infections.

**Table 1:** Cost estimates per month (during surveillance) and per case (during an outbreak) to prevent and control dengue fever introductions in Cairns, Australia.

Item	Cost units	Cost (2009 U$)
Surveillance		
Dengue action response team (DART)^1^	per month	25,967
Control^2^		
Personnel	per case	1,336
Travel	per case	282
Vehicle use	per case	64
Insecticides	per case	279
Miscellaneous expenses	per case	177
Diagnosis		
Diagnosis tests^3^	per case	96
Blood bank screening^4^	per case	805
Days lost due to disease^5^	per case	508

1 DART's responsibility is to implement mosquito prevention and control. In large outbreaks DART is supplemented by environmental health and municipal agents.

2 Vector control encompasses selective indoor insecticide residual spraying (SC 2.5% lambda-cyhalothrin, Demand) and larval control/source reduction activities (removal of small containers and treatment of large containers with S-methophene pellets or residual surface sprays) in premises within 100 meters of a case.

3 Serum samples are forwarded to the reference laboratory where they are screened for the presence of anti-dengue IgM and IgG using a combined pool of flavivirus antigens in capture ELISA assays. Positive IgM samples are further analyzed using flavivirus-specific IgM ELISA capture assays in order to identify the serotype of the infecting dengue virus. Additionally, real-time TaqMan reverse transcriptase-polymerase chain reaction is performed to detect dengue virus RNA.

4 Information provided by the Australian Red Cross Blood Service.

5 Each cased was assumed to loose, on average, 5 work days. Daily costs were estimated by dividing the median monthly income in Cairns (US$ 25,419; source: Australian Bureau of Statistics) by the number of working days (250).

## Results and Discussion

The dengue outbreaks in Cairns demonstrate the vulnerability of developed countries to mosquito-borne pathogens that are major international public health concerns [7]. Our analysis shows that delaying control responses translates into an exponential increase in both the number of human cases and health costs (Figure 1). The cumulative cost of a strategy with active surveillance and *res* of 2 was US$ 0.15 and US$1.1 million for 2003 and 2009 epidemics, respectively (Figure 1). Responding to the same outbreaks 4–6 weeks later (*res* = 6–8) would have resulted in cumulative costs of containing the 2003 and 2009 outbreaks that are 86 (or US$ 13 million; Figure 1 C) and 346 (or US$382 million; Figure 1 D) times as high, respectively, than a strategy based on ongoing active surveillance. By the 9^th^ week of an outbreak the costs accrued in controlling it increased exponentially and far surpassed the costs of a strategy with sustained surveillance and early case detection (*res* = 2) (Figure 1 C–D). Thus, a delayed reaction to both Cairns dengue outbreaks would have resulted in drastically escalated total costs of up to US$ 382 million. Indeed, a slight difference in the virulence of the invading strain (Δ*R_0_* = 0.1 between outbreaks) would have increased total costs by one order of magnitude (Figure 1 C–D). Notably, our predictions show that the costs to contain the 2009 outbreak in a city with a climate comparable to Miami, but with <10% of the population, would have been an order of magnitude higher than the proposed CDC budget cut that will impact the whole US.

Without a strong human and vector surveillance system, detection and response to emerging vector-borne diseases that can present, in many instances, undetermined symptoms in humans could be severely impaired. The emergence of WNV in New York City in 1999 is a clear example of the consequences that a delayed response can have on the outcome of a novel arboviral introduction [8], [9]. The first glimpse of WNV transmission occurred with the notification of unusual bird deaths in late June. The incorrect diagnosis of a cluster of human cases as St. Luis encephalitis in late August prompted the initiation of vector control actions, almost 2 months since the detection of bird deaths [8], [9]. By the time vector control was in place and WNV confirmed as the putative source of human and bird infections, the infection could not be contained (particularly in the bird population), and subsequently progressed throughout the US, generating 28,961 WNV human cases and 1,130 fatalities by the end of 2008 [10]. Vector-borne disease surveillance in the U.S. improved significantly after this failure to contain WNV [11]. A strong network of state and local health departments rely on CDC funds for personnel and routine seasonal testing of mosquitoes for WNV and other viruses. Indeed, one of the reasons the recent emergence of dengue in mainland US (after a 50-year hiatus [12]) was rapidly detected and contained is the through the presence in Florida of the CDC-supported vector surveillance network.

Without CDC funds, mosquito testing would be halted, and detection of transmission events or novel viral introductions significantly delayed (with response delayed by even more than 8 weeks), turning CDC into a reactive rather than preventive health service. Our analysis clearly shows that the total costs of preparedness through surveillance are far lower than the ones needed to respond to the introduction of vector-borne pathogens, even without consideration of the cost in human lives and well-being. Our economic analysis provides strong ammunition from an ethical, economic and scientific standpoint for lawmakers to retain the investments in this cost-effective preventive public health strategy. In fact, our analysis points to the need for more, rather than less, funding for vector-borne disease surveillance. The probability for early detection of an introduction of a vector borne disease agent, or for rapid interruption of transmission if an outbreak were to occur, are a direct function of adequate funding for vector borne disease research and surveillance.
